# Tonoplast-localized nitrate uptake transporters involved in vacuolar nitrate efflux and reallocation in *Arabidopsis*

**DOI:** 10.1038/s41598-017-06744-5

**Published:** 2017-07-25

**Authors:** Ya-Ni He, Jia-Shi Peng, Yao Cai, De-Fen Liu, Yuan Guan, Hong-Ying Yi, Ji-Ming Gong

**Affiliations:** 1 National Key Laboratory of Plant Molecular Genetics and National Center for Plant Gene Research (Shanghai), CAS Center for Excellence in Molecular Plant Sciences, Institute of Plant Physiology and Ecology, Shanghai Institutes for Biological Sciences, Chinese Academy of Sciences, Shanghai, 200032 People’s Republic of China; 2 University of Chinese Academy of Sciences, Beijing, 100049 China; 3 Forestry and Fruit Tree Research Institute, Shanghai Academy of Agricultural Sciences, Shanghai, 201403 China

## Abstract

A great proportion of nitrate taken up by plants is stored in vacuoles. Vacuolar nitrate accumulation and release is of great importance to nitrate reallocation and efficient utilization. However, how plants mediate nitrate efflux from vacuoles to cytoplasm is largely unknown. The current study identified NPF5.11, NPF5.12 and NPF5.16 as vacuolar nitrate efflux transporters in *Arabidopsis*. Histochemical analysis showed that *NPF5.11*, *NPF5.12* and *NPF5.16* were expressed preferentially in root pericycle cells and xylem parenchyma cells, and further analysis showed that these proteins were tonoplast-localized. Functional characterization using cRNA-injected *Xenopus laevis* oocytes showed that NPF5.11, NPF5.12 and NPF5.16 were low-affinity, pH-dependent nitrate uptake transporters. In *npf5.11 npf5.12 npf5.16* triple mutant lines, more root-fed ^15^NO_3_^−^ was translocated to shoots compared to the wild type control. In the *NPF5.12* overexpression lines, proportionally less nitrate was maintained in roots. These data together suggested that NPF5.11, NPF5.12 and NPF5.16 might function to uptake nitrate from vacuoles into cytosol, thus serving as important players to modulate nitrate allocation between roots and shoots.

## Introduction

Nitrate is the major nitrogen source for most plants, especially those grown in aerobic soil conditions^1^. Once taken up from soil, nitrate is either assimilated or stored in vacuoles. As the largest organelle in fully expanded plant cells, vacuoles are identified as the major nitrate storage pools and contain up to 90% of the total cellular nitrate^2, 3^. However, vacuolar nitrate is not readily accessible to NR (nitrate reductase), thus it has to be reallocated for metabolic use when necessary^4, 5^. Vacuolar nitrate release helps to maintain the relative steady level of cytosolic nitrate when external nitrogen supply was limited^6, 7^. During the dark-to-light transition, nitrate remobilization from vacuoles was also observed to comply with the new steady state caused by the increased NR activity^4^. Therefore, vacuolar nitrate and its remobilization are important for the regulation of nitrogen assimilation and nitrogen use efficiency^5, 8, 9^.

Transport across the tonoplast is energized by the vacuolar H^+^-ATPase (V-ATPase) and the vacuolar H^+^-pyrophosphatase (V-PPase), which create the proton gradient and the membrane potential^10–12^. For nitrate, its accumulation in vacuoles is probably mediated by the nitrate/proton antiport machinery^13–16^ and the nitrate/proton symport system may serve to remobilize vacuolar nitrate^16–18^. However, only a few tonoplast localized nitrate transporters have been identified up to date. AtCLCa and AtNRT2.7 are two transporters responsible for nitrate accumulation in vacuoles^19–21^. AtCLCa was a tonoplast localized 2NO_3_^−^/1 H^+^ antiporter expressed in both shoots and roots^20, 22^. Disruption of AtCLCa led to approximately 50% decrease of vacuolar nitrate, suggesting an important role for AtCLCa in vacuolar nitrate accumulation^19, 20^. AtNRT2.7, however, was a tonoplast localized transporter expressed exclusively in seeds, which regulated the kinetics of seed germination by affecting nitrate storage in seed vacuoles^21^. AtCLCc was also supposed to be involved in vacuolar nitrate accumulation, because it was tonoplast localized and the related mutants showed lower nitrate contents^22, 23^. Regarding nitrate efflux from vacuoles, however, indirect evidences imply that AtCLCb and OsNPF7.2 might get involved, as they both were tonoplast-localized, and heterologous expression in *Xenopus laevis* oocytes indicated that they mediated nitrate uptake, but no evidence showed that functional disruption of these genes led to nitrate accumulation in vacuoles^24, 25^. AtCLCa was also implied to get involved in vacuolar nitrate efflux, because it mediated anion homeostasis in stomata movement^26^, while nitrate is one of the anions contributing to stomatal movement^27, 28^.

In the current study, three tonoplast-localized NRT1/NPF family members NPF5.11, NPF5.12 and NPF5.16 were identified by bioinformatics analysis, and functional characterization was performed. Our data suggested that these three transporters were all tonoplast localized, and mediated nitrate uptake in a pH-dependent low-affinity manner when heterologously expressed in oocytes. Further analysis indicated that they possibly modulated nitrate allocation between roots and shoots via vacuolar nitrate release.

## Results

### Tonoplast Localization of NPF5.11, NPF5.12 and NPF5.16

**Figure 1:**
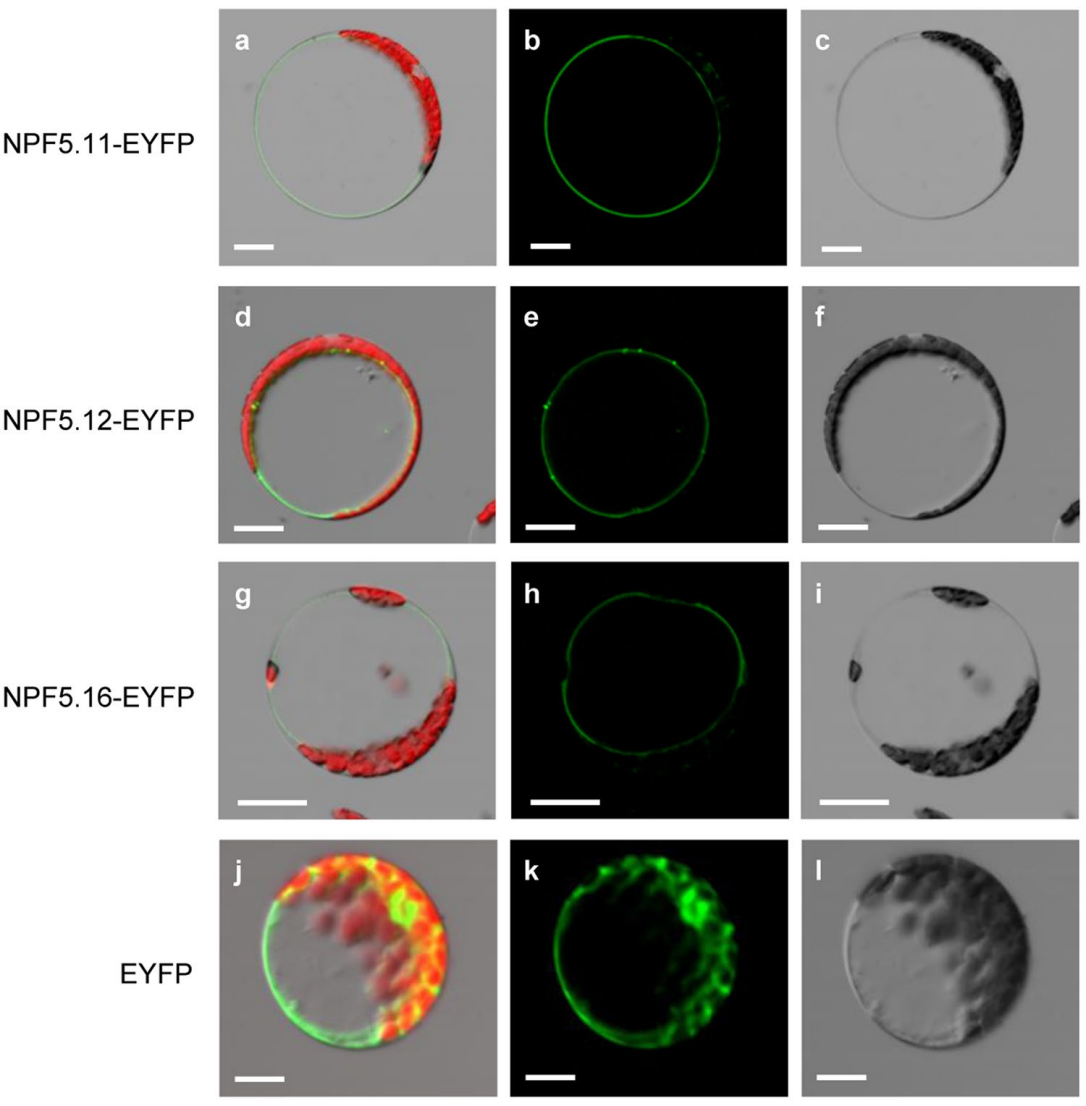
Subcellular localization of NPF5.11, NPF5.12 and NPF5.16. *NPF5.11-EYFP* (**a**–**c**), *NPF5.12-EYFP* (**d**–**f**), *NPF5.16-EYFP* (**g**–**i**) or *EYFP* (**j**–**l**) was driven by the cauliflower mosaic virus 35 S promoter and transiently expressed in *Arabidopsis* mesophyll protoplasts. Overlap images of EYFP (green) and chlorophyll (red) fluorescence (**a**,**d**,**g**,**j**), EYFP fluorescence (**b**,**e**,**h**,**k**), and bright-field (**c**,**f**,**i**,**l**) images are shown. Bars = 20 μm.

Based on previous studies about vacuole proteome^29^, we targeted NPF5.12 and its close homologs NPF5.11 and NPF5.16, members of NRT1/NPF family^30^, as candidate transporters for vacuolar nitrate transport. NPF5.11, NPF5.12 and NPF5.16 were predicted to contain 11, 12, 10 transmembrane domains (http://www.cbs.dtu.dk/services/TMHMM/), respectively. To investigate the subcellular localization of NPF5.11, NPF5.12 and NPF5.16, *NPF5.11*-*EYFP*, *NPF5.12*-*EYFP* and *NPF5.16*-*EYFP* driven by the cauliflower mosaic virus 35 S promoter were transiently expressed in *Arabidopsis* mesophyll protoplast. The yellow fluorescence signals of NPF5.11-EYFP (Fig. 1a–c), NPF5.12-EYFP (Fig. 1d–f) and NPF5.16-EYFP (Fig. 1g–i) were detected in the membrane around the large central vacuole. Similar results were obtained by transiently expressing these fusion proteins in onion epidermal cells, verifying that NPF5.11, NPF5.12 and NPF5.16 were tonoplast localized (Supplementary Fig. S1).

### NPF5.11, NPF5.12 and NPF5.16 are pH-dependent Low-Affinity Nitrate Transporters

Given that *Xenopus laevis* oocytes did not contain vacuoles, we firstly tested the expression and localization of NPF5.11, NPF5.12 and NPF5.16 in oocytes. As a well-documented nitrate transporter^31^, NRT1.8 fused with GFP was used as a positive control and its fluorescence was detected at the rim of oocytes (Supplementary Fig. S2). Likewise, NPF5.11-EYFP, NPF5.12-EYFP and NPF5.16-EYFP fusion proteins could express in plasma membrane of oocytes though they were tonoplast localized transporters in *Arabidopsis*, indicating that we could use oocytes to explore the function of NPF5.11, NPF5.12 and NPF5.16 (Supplementary Fig. S2).

**Figure 2:**
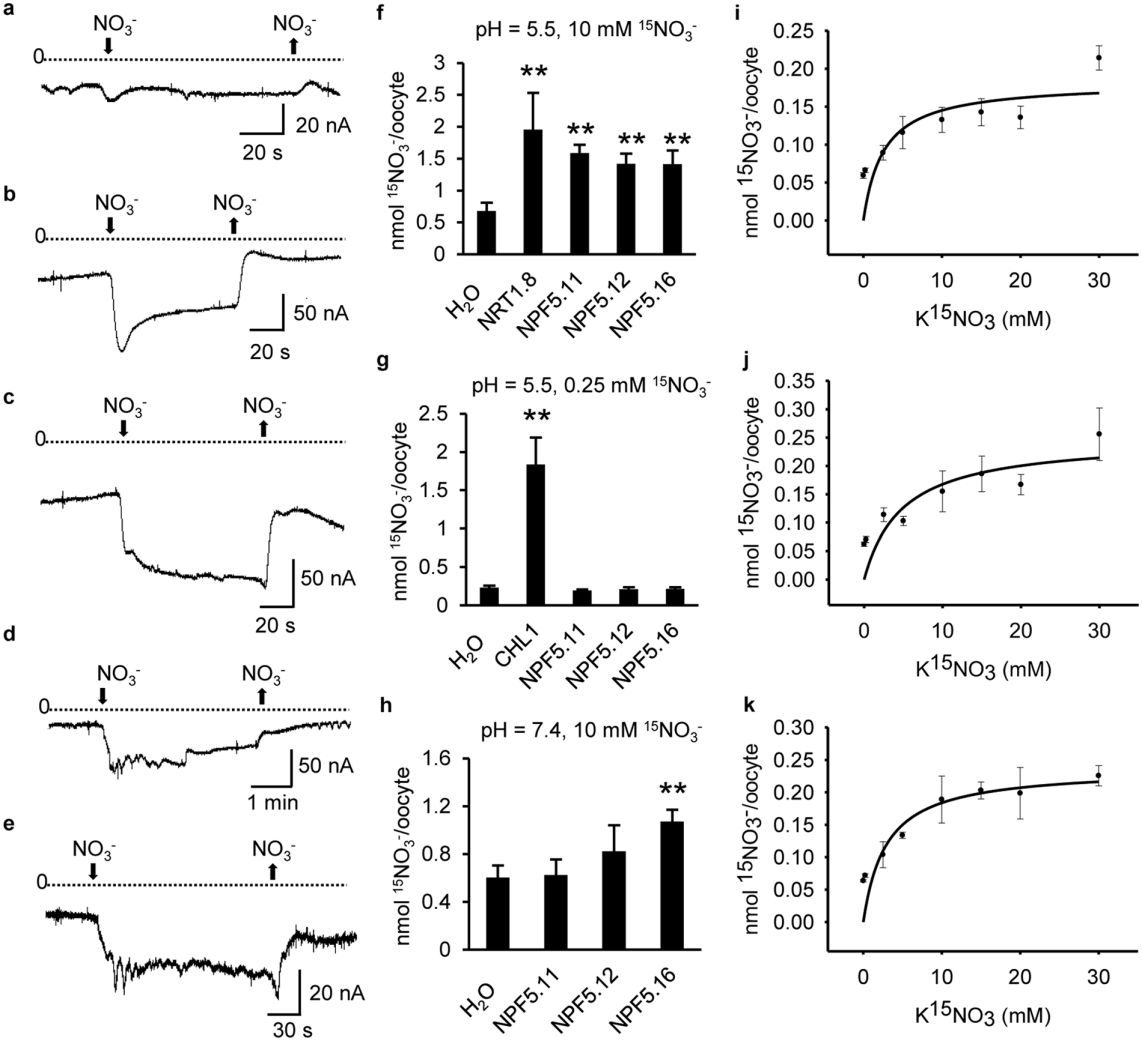
Functional characterization of NPF5.11, NPF5.12 and NPF5.16 in oocytes. (**a**–**e**) Currents elicited in oocytes injected with H_2_O (**a**), *CHL1* cRNA (**b**), *NPF5.11* cRNA (**c**), *NPF5.12* cRNA (**d**) or *NPF5.16* cRNA (**e**). Oocytes were voltage clamped at −60 mV and representative inward currents elicited by 10 mM NO_3_^−^ at pH 5.5 were recorded. (**f**–**h**) Nitrate uptake activity in oocytes injected with H_2_O, *NPF5.11* cRNA, *NPF5.12* cRNA, *NPF5.16* cRNA, *NRT1.8* cRNA or *CHL1* cRNA. Oocytes were incubated with 10 mM ^15^NO_3_^−^ at pH 5.5 (**f**), 0.25 mM ^15^NO_3_^−^ at pH 5.5 (**g**) or 10 mM ^15^NO_3_^−^ at pH 7.4 (**h**) for 12 h. Values are means ± SD (n = 8–12). Asterisks indicate difference at *P* < 0.01 (**) compared with the H_2_O-injected oocytes by Student’s *t*-test. (**i**–**k**) Uptake kinetics of NPF5.11 (**i**), NPF5.12 (**j**) and NPF5.16 (**k**). Oocytes injected with *NPF5.11* cRNA (**i**), *NPF5.12* cRNA (**j**) or *NPF5.16* cRNA (**k**) were incubated with indicated concentrations of ^15^NO_3_^−^ at pH 5.5 for 1.5 h, and the ^15^N contents were determined. Values are means ± SD (n = 6–12). The *K*m was calculated by fitting to the Michaelis-Menten equation using a nonlinear least squares method in the SigmaPlot program. The *K*m was 2.57 mM, 4.84 mM, or 2.91 mM for NPF5.11, NPF5.12 or NPF5.16, respectively.

Electrophysiological analysis using cRNA-injected oocytes were performed to test whether NPF5.11, NPF5.12 and NPF5.16 could use nitrate as substrate. After 2 days of incubation, oocytes were voltage clamped at −60 mV and perfused with 10 mM nitrate at pH 5.5. Compared with water-injected oocytes (Fig. 2a), a larger inward current was induced by CHL1-injected oocytes (Fig. 2b), as reported before^32^. NPF5.11-, NPF5.12- or NPF5.16-injected oocytes also induced inward currents (Fig. 2c–e), indicating that they were electrogenic transporters using nitrate as the substrate.

Nitrate transport activities of NPF5.11, NPF5.12 and NPF5.16 were further confirmed by analyzing ^15^NO_3_^−^ uptake activity. NPF5.11-, NPF5.12-, NPF5.16- or NRT1.8-injected oocytes showed enhanced ^15^NO_3_^−^ uptake activity when incubated with 10 mM ^15^NO_3_^−^ at pH 5.5, compared with water-injected oocytes (Fig. 2f). However, NPF5.11-, NPF5.12- or NPF5.16-injected oocytes almost did not uptake ^15^NO_3_^−^ when assayed with 0.25 mM ^15^NO_3_^−^ at pH 5.5, while CHL1-injected oocytes still showed high uptake activity (Fig. 2g). In addition, as expected for proton-coupled transporters, ^15^NO_3_^−^ uptake activities of NPF5.11-, NPF5.12- or NPF5.16-injected oocytes at pH 7.4 were much lower than those at pH 5.5, comparable with the negative control (Fig. 2h). It is worth mentioning that NPF5.11, NPF5.12 and NPF5.16 did not efflux nitrate from oocytes under pH 5.5 or pH 7.4 (Supplementary Fig. S3).

To further determine the uptake affinity of NPF5.11, NPF5.12 and NPF5.16, uptake activity of NPF5.11-, NPF5.12- or NPF5.16-injected oocytes at pH 5.5 was measured using different concentrations of ^15^NO_3_^−^ ranging from 0.25 mM to 30 mM as substrates. The *K*_m_ for nitrate was calculated by fitting to the Michaelis-Menten equation, and was estimated as 2.57 mM, 4.84 mM, 2.91 mM respectively for NPF5.11, NPF5.12 and NPF5.16 (Fig. 2i–k). Taken together, these results suggested that NPF5.11, NPF5.12 and NPF5.16 were pH-dependent low-affinity nitrate transporters.

### *NPF5.11*, *NPF5.12* and *NPF5.16* are mainly expressed in vascular stele of roots and leaves

**Figure 3:**
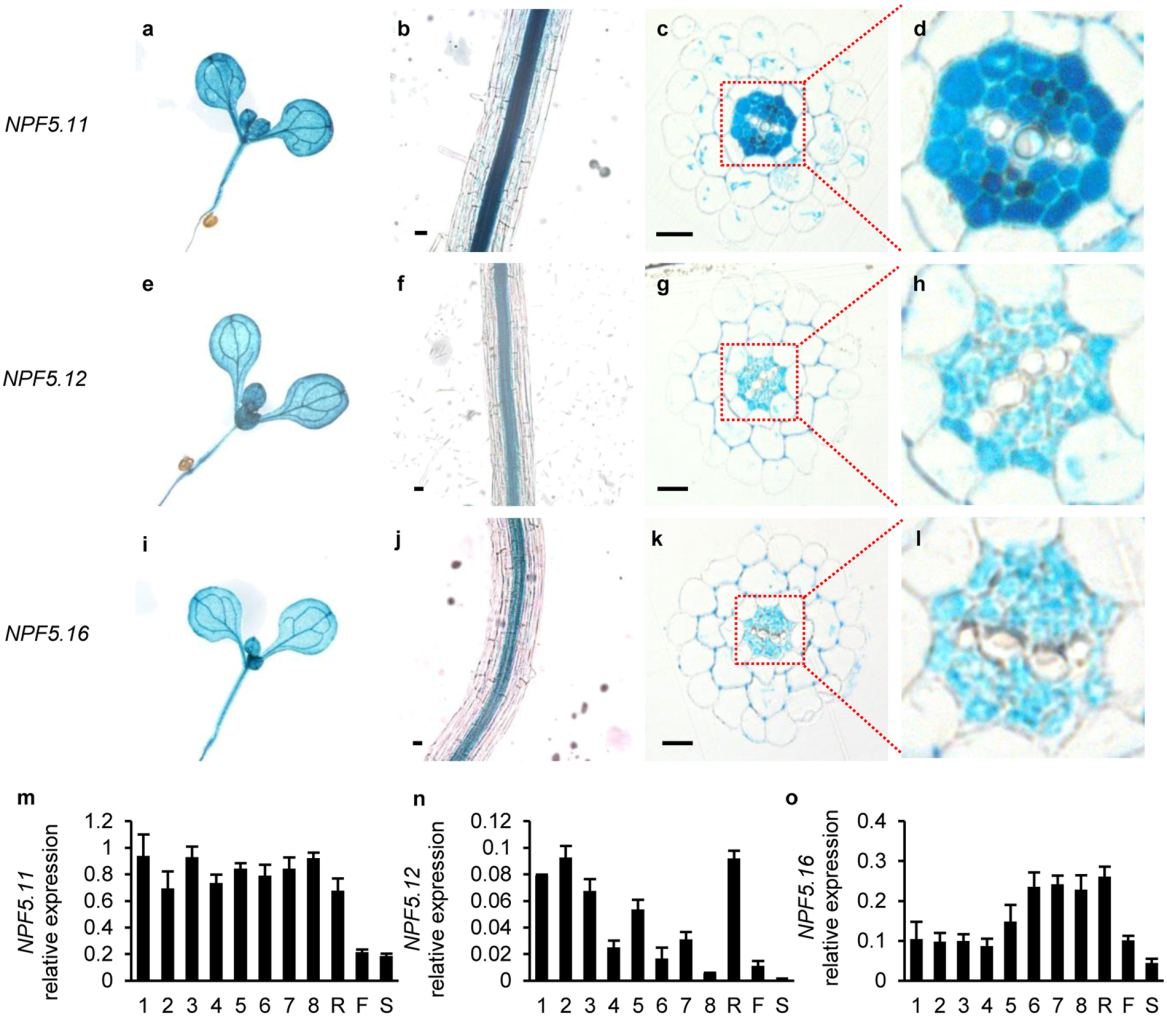
*NPF5.11*, *NPF5.12* and *NPF5.16* are preferentially expressed in vascular tissues. Histochemical localization of GUS activity in *NPF5.11*_*pro*_::GUS transgenic plants (**a**–**d**), *NPF5.12*_*pro*_::GUS transgenic plants (**e**–**h**) and *NPF5.16*_*pro*_::GUS transgenic plants (**i**–**l**). The expression patterns of *NPF5.11*, *NPF5.12* and *NPF5.16* were determined in whole-mount seedlings (**a**,**e**,**i**), seedling roots (**b**,**f**,**j**) or cross-sectioned seedling roots **(c,d,g,h,k,l**). (**m**,**n**,**o**) Transcript expression of *NPF5.11* (**m**), *NPF5.12* (**n**) and *NPF5.16* (**o**) in 28 d old plants. 1–8 indicated leaf positions arranged according to leaf ages (old to young); R, root; F, flower; S, stem. Data were normalized to that of *SAND*. Values are means ± SD, n = 3. Bars = 10 μm.

The tissue localization of genes could provide hint for their physiological role. To elucidate the expression pattern of *NPF5.11*, *NPF5.12* and *NPF5.16*, promoter-GUS (β-glucuronidase) reporter analysis was performed. The promoter region of *NPF5.11*, *NPF5.12* and *NPF5.16* were used for driving the expression of GUS in Columbia (Clo-0). As shown in Fig. 3, *NPF5.11*, *NPF5.12* and *NPF5.16* had a similar expression pattern, expressing in both shoots and roots. In shoots, they were mainly expressed in leaf veins while the mesophyll cells were also stained (Fig. 3a,e,i). In roots, GUS activity was detected in root vascular stele (Fig. 3b,f,j). Cross-sections of young seedling roots showed that *NPF5.11*_*pro*_::*GUS*, *NPF5.12*_*pro*_::*GUS* and *NPF5.16*_*pro*_::*GUS* were expressed in pericycle cells and parenchyma cells, and *NPF5.11*_*pro*_::*GUS* was also expressed in the phloem (Fig. 3c,d,g,h,k,l).

The expression patterns of these three genes in adult plant were further investigated by qRT-PCR analysis. *NPF5.11*, *NPF5.12* and *NPF5.16* all showed high expression in root while the expression in flower and stem was quite low (Fig. 3m,n,o). The expression of *NPF5.12* in old leaves was higher than that in young leaves, while *NPF5.16* was preferentially expressed in young leaves (Fig. 3n,o).

### More nitrate is translocated to shoots in triple mutant

**Figure 4:**
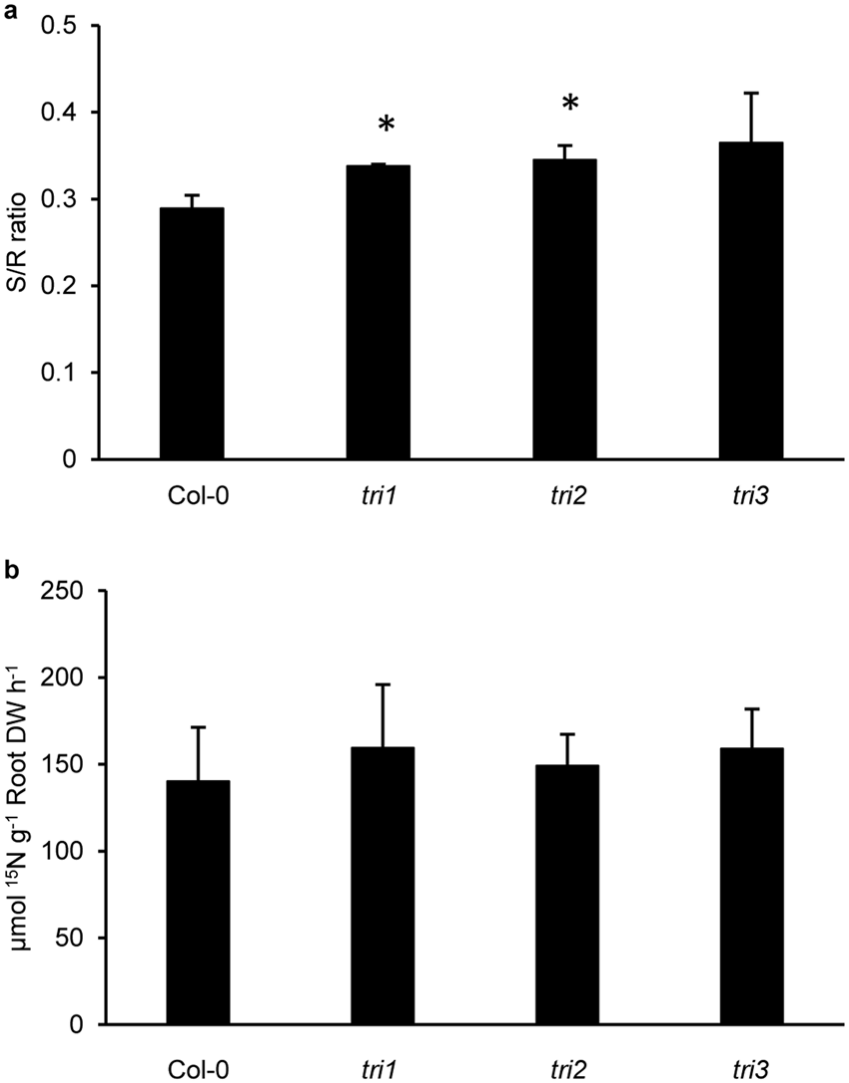
Root-to-shoot nitrate transport enhanced in the triple mutant plants *npf5.11 npf5.12 npf5.16*. Plants were grown in hydroponics for 28 days and treated with 2.25 mM K^15^NO_3_ for 30 min. ^15^N contents in shoots and roots were analyzed. ^15^N concentration ratio between shoots and roots (S/R ratio, **a**) and root uptake activity (**b**) were determined. Values are means ± SD, n = 3. Asterisks indicate difference between wild type and triple mutant lines at *P* < 0.05 (*) by Student’s *t*-test.

Considering the tonoplast localization (Fig. 1) and pH-dependent nitrate uptake (Fig. 2), we proposed that NPF5.11, NPF5.12 and NPF5.16 might be responsible for uptaking nitrate from vacuole (pH 5.5^33^) to cytoplasm in *Arabidopsis*. To test this hypothesis, we generated several lines of their single, double, and even triple mutants (Supplementary Figs S4,S6,S7). Note that only double mutant lines of *npf5.12 npf5.16* were generated because *NPF5.11* and *NPF5.12* were tightly linked in *Arabidopsis* genome and *npf5.11* mutant lines were in Ws background. Given vacuolar nitrate efflux is supposed to be enhanced when nitrogen is limited, we firstly analyzed the nitrate contents in leaves and roots in these mutants under both control condition and nitrogen-starved condition. As shown in Supplementary Figs S5, S6 and S7, no obvious difference was observed between the wild type control and all the mutants. However, when they were fed with ^15^NO_3_^−^, the ratio of ^15^N concentration in shoots against that in roots (shoot/root) was higher in triple mutant lines than in the wild type (Fig. 4a, Supplementary Fig. S8), while no significant difference was observed between the single mutant lines and the wild type (Supplementary Fig. S9). These results suggested that more ^15^NO_3_^−^ was translocated to shoots in triple mutants, while our data also indicated that the root uptake capacity of triple mutant lines was not affected (Fig. 4b).

### Root nitrate content is reduced in *NPF5.12* overexpression lines

**Figure 5:**
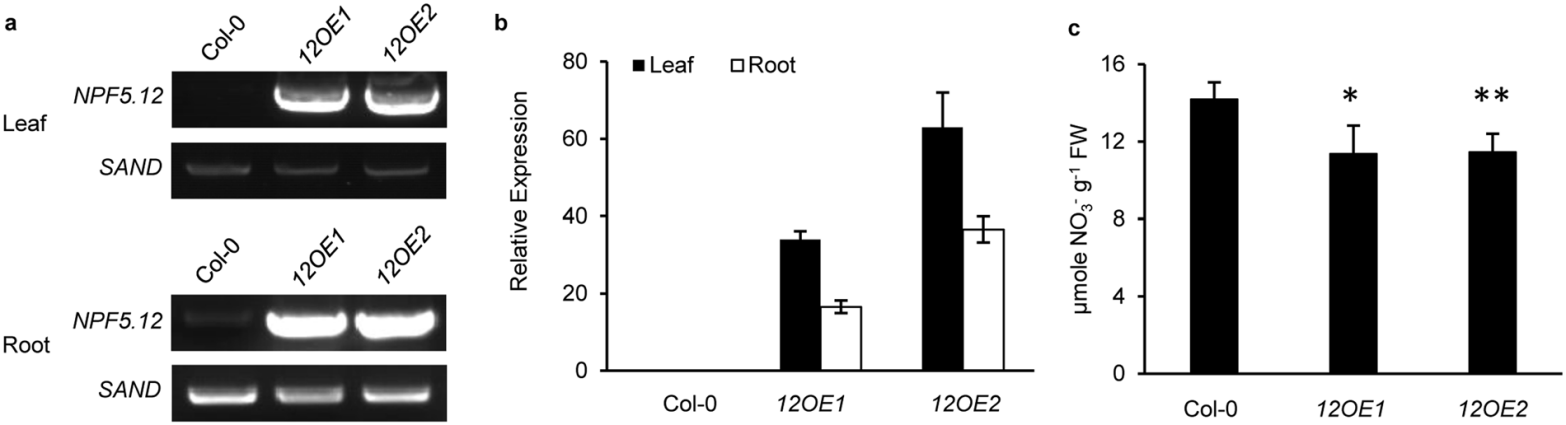
Decreased nitrate accumulation in roots of *NPF5.12* overexpression lines. (**a**,**b**) Identification of *NPF5.12* overexpression lines by RT-PCR (**a**) and quantitative PCR analysis (**b**). (**c**) 24 days old hydroponically grown plants were subjected to nitrogen-starvation for 30 h, then roots were sampled and nitrate contents were determined by HPLC. *12OE1* and *12OE2* were two independent *NPF5.12* overexpression lines. Values are means ± SD, n = 5–7. Asterisks indicate difference between wild type and overexpression lines at *P* < 0.05 (*) and *P* < 0.01 (**) by Student’s *t*-test.

To further investigate the function of NPF5.11, NPF5.12 and NPF5.16, the overexpression lines of *NPF5.11*, *NPF5.12* and *NPF5.16* under the control of 35 S promoter were generated (Fig. 5a,b) and the nitrate content was analyzed (Fig. 5c, Supplementary Figs S10, S11). The result showed that nitrate contents in roots of *NPF5.12* overexpression lines were lower than that of wild type under nitrogen-starved condition (Fig. 5c). This observation was not found in *NPF5.11* and *NPF5.16* overexpression lines (Supplementary Fig. S11). One explanation could be that NPF5.11 and NPF5.16 might require other components to function properly in planta.

## Discussion

Significant progresses have been made in clarifying the nitrate uptake and transport in *Arabidopsis* by the characterization of the transporters in NRT1/NPF, NRT2, CLC and SLAC/SLAH families^34^. However, our current knowledge about nitrate transport across the tonoplast is quite limited though the significance of this process is widely recognized.

The transporters responsible for vacuolar nitrate efflux should be tonoplast-localized and uptake nitrate toward cytoplasm. Our data suggested that NPF5.11, NPF5.12 and NPF5.16 were localized in tonoplast in *Arabidopsis* (Fig. 1) and plasma membrane in *Xenopus laevis* oocytes (Supplementary Fig. S2), and in oocytes they could elicit inward currents by external nitrate and uptake nitrate in a pH dependent way (Fig. 2). When these results were assigned to the topology of the plant tonoplast, the inward currents might represent NO_3_^−^/H^+^ efflux from the vacuole to the cytoplasm^24^, because the external medium for oocytes corresponds to the vacuole in planta^35, 36^ and the pH of vacuole in *Arabidopsis* is about 5.5^33, 37^. Thus we speculated that NPF5.11, NPF5.12 and NPF5.16 were responsible for vacuolar nitrate release in *Arabidopsis*.

Considering that NPF5.11, NPF5.12 and NPF5.16 are predominantly expressed in vacuole membrane of pericycle cells and xylem parenchyma cells in roots (Figs 1, 3). We proposed that they might be involved in the regulation of nitrate long-distance transport by modulating the vacuolar sequestration capacity (VSC) of nitrate in roots. Relationship between VSC and long-distance transport of metals in plant have been well documented^38^, and accumulating evidences indicated that VSC of essential anions including sulfate and nitrate also regulated their long-distance transport^39, 40^. The vacuolar sulfate efflux transporters SULTR4;1 and SULTR4;2 played an essential role in delivering sulfate to the xylem vessels by balancing storage and turnover of sulfate in the root vacuoles^39^. While Han *et al*., found that the decreased VSC of nitrate in roots would enhance nitrate transport to shoots and contribute to a higher nitrogen use efficiency (NUE)^40^. Our hypothesis about the physiological role of NPF5.11, NPF5.12 and NPF5.16 was supported by the observation that more proportion of ^15^NO_3_^−^ was translocated to shoots in triple mutant lines (Fig. 4) and overexpression of *NPF5.12* resulted in a lower nitrate contents in roots (Fig. 5c). In the triple mutant lines, root VSC increased but not too much was available to newly absorbed nitrate due to the impaired nitrate efflux from vacuoles, resulting in less vacuolar nitrate sequestration and the consequent enhancement of ^15^NO_3_^−^ long-distance transport to shoots when fed with ^15^NO_3_^−^ for a short time (30 min). In *NPF5.12* overexpression lines, the overall nitrate contents in roots decreased because of the lower VSC of nitrate, thus leading to the higher S/R ratios in the overexpression lines (Fig. 5c, Supplementary Fig. S10b).

No significant difference of nitrate contents was detected between all the mutants and wild type under various growth conditions we tested (Supplementary Figs S5, S6, S7). We proposed that there might be other transporters or channels that function redundantly with NPF5.11, NPF5.12 and NPF5.16, as the reutilization of vacuolar nitrate is crucial to environmental adaption for plants. The speculation is according with the observation that more ^15^NO_3_^−^ was translocated to shoots in triple mutant but not in single mutant. Similarly, no obvious changes in nitrate allocation was observed in mutants of *AtCLCb* or *OsNPF7.2* ^24, 25^. In addition, considering their specific tissue localization in roots (Fig. 3), we speculated that physiological effect of NPF5.11, NPF5.12 and NPF5.16 in nitrate allocation might be more noticeable specifically in pericycle cells and parenchyma cells. Thus more definitive evidences are needed in the future to demonstrate the working model for vacuolar nitrate efflux.

## Methods

### Plant Materials and Growth Conditions

*Arabidopsis* (*Arabidopsis thaliana*) ecotype Col-0 or Ws was used as the wild-type control. The *Arabidopsis* T-DNA mutant lines *npf5.11-1* (FLAG_493A07) and *npf5.11-2* (FLAG_592C02) were ordered from INRA (National Institute for Agricultural Research)^41^; *npf5.12-1* (GABI_810C10) was ordered from NASC (European Arabidopsis Stock Centre)^42^; *npf5.12-2* (CS871745), *npf5.16-1* (SALK_152449 C) and *npf5.16-2* (SALK_200474 C) were ordered from ABRC (Arabidopsis Biological Resource Center)^43, 44^. Homozygous mutant plants were screened by PCR^45^. The *npf5.12 npf5.16* double mutant lines of were generated by crossing *npf5.12* and *npf5.16* and identified by PCR. The double mutant *npf5.12 npf5.16* was further transformed with CRISPR/Cas9 system to generate the triple mutant *npf5.11 npf5.12 npf5.16*, using two different target sequences^46^. The double mutant lines used for transformation were: *tri1*, *npf5.12-1 npf5.16-2*; *tri2*, *npf5.12-1 npf5.16-1*; *tri3*, *npf5.12-2 npf5.16-1*. The CRISPR-Cas9 T-DNA was not existent in triple mutants by *Cas9* PCR confirming^47^. The primers used in these assays are listed in Supplemental Table S1.

*Arabidopsis* plants were grown in quarter-strength hydroponic solution at 22 °C with 16-h-light/8-h-dark cycles as described^48^. Plants were grown to 3-4 weeks old and then were treated with nitrogen-starved nutrient solution by replacing KNO_3_ and Ca(NO_3_)_2_ with KCl and CaCl_2_ as indicated time.

### Functional Analysis of NPF5.11, NPF5.12 and NPF5.16 in *Xenopus laevis* Oocytes

cDNA fragments of targeted genes were recovered by restriction digestion and then subcloned into the oocyte expression vector pOO2^49^. cRNA was synthesized using the Ambion mMessage mMachine kit according to the manufacturer’s manual. Oocytes were isolated and injected with 50 ng cRNA as described previously^50^. *CHL1* cRNA or *NRT1.8* cRNA injected oocytes were used as positive control and water-injected oocytes were used as negative control. Oocytes were incubated in a ND-96 Ringer solution for 2 days as described^31^. Voltage clamp recordings were initiated in a bath solution containing 230 mM mannitol, 0.15 mM CaCl_2_, and 10 mM MES/Tris, pH 5.5^51^. Nitrate uptake or efflux assays with ^15^NO_3_^−^ were performed as described^50, 52, 53^ using a continuous-flow isotope ratio mass spectrometer coupled to a carbon nitrogen elemental analyzer (Vario EL III/Isoprime; Elementar). Uptake kinetics assays were performed as described^54^.

### EYFP Fusion and Subcellular Localization

The amplified *NPF5.11*, *NPF5.12* and *NPF5.16* cDNA fragments were cloned in frame in front of *EYFP* in the vector 35 S::*EYFP*/PA7, resulting in the *NPF5.11-EYFP*, *NPF5.12-EYFP* and *NPF5.16-EYFP* constructs under the control of the 35 S promoter. The resulted constructs were transiently expressed in *Arabidopsis* protoplast using the polyethylene glycol-mediated transformation method^55^. Alternatively, these constructs were also transiently expressed in onion epidermal cells using a particle gun–mediated system (PDS-1000/He; Bio-Rad). The transformed protoplasts and bombarded cells were held in the dark at 22 °C for more than 30 h followed by EYFP imaging using confocal microscopy (Olympus-FV1000).

For the GFP- or EYFP- fusion proteins expression in oocytes assay, the constructs were generated by introducing *NRT1.8-GFP*, *NPF5.11-EYFP*, *NPF5.12-EYFP* or *NPF5.16-EYFP* into the vector pOO2. The cRNA was synthesized and injected into oocytes. After cultivating 2 days, fluorescence was observed using confocal microscope (Olympus-FV1000).

### Histochemical Analysis and Tissue Sectioning

A 1679-bp, a 1314-bp or a 1695-bp genomic fragment immediately upstream from the *NPF5.11*, *NPF5.12* or *NPF5.16* start codon, respectively, was amplified using primers listed in Supplemental Table S1. After sequencing, the fragments were cloned into the binary vector GUS /pCambias1300 and were then transformed into Col-0 as described^31^. GUS staining was performed overnight as described^31^. Semithin sections (4μm) were cut, mounted on glass slides, and visualized on Leica-DM6000.

### RT-PCR and Quantitative RT-PCR

Plants were grown to 28 days old in hydroponics, and then were sampled as indicated. Total RNA was extracted using TRIzol reagent (Invitrogen). First-strand cDNA synthesis and RT-PCR were performed as described^31^. Quantitative RT-PCR was performed on a Corbett Research Rotor-Gene 3000 thermal cycler using SYBR Premix Ex-Taq (TaKaRa) according to the manufacturer’s protocol. The primers used in these assays are listed in Supplemental Table S1, and the expression levels were normalized to those of the *SAND* or *Actin2* control.

### Nitrate Content Determination by HPLC

Plants were grown to 3–4 weeks old and were treated with nitrogen-starved nutrient solution for indicated time. Leaves and roots were harvested and washed at least three times by ultrapure water for 5 min and then extracted nitrate as described^56^.

### Analysis of Root-to-Shoot Nitrate Transport Using ^15^NO_3_^−^

Wild type and triple mutant plants were grown in hydroponics for 28 days old and then were transferred to 0.1 mM CaSO_4_ for 1 min, labeled in quarter-strength hydroponics medium with 2.25 mM K^15^NO_3_ with 99% atom excess of ^15^N for 30 min. At the end of labeling, plants were washed in 0.1 mM CaSO_4_ for 1 min and the shoots and roots were separated. The shoots and roots were sampled and detected as described^57^.

### Statistical Analysis

Two-tailed Student’s *t* tests were performed. Differences were deemed significant (*) at P < 0.05 and extremely significant (**) at P < 0.01.

### Data Availability

The datasets generated during and/or analyzed during the current study are available from the corresponding author on reasonable request.

## Electronic supplementary material

Supplementary Figure and Table
