# Supplementary material for: Tonoplast-localized nitrate uptake transporters involved in vacuolar nitrate efflux and reallocation in Arabidopsis
Source: Sci Rep. 2017 Jul 25;7:6417. doi: 10.1038/s41598-017-06744-5 (PMC5526873; doi:10.1038/s41598-017-06744-5)
Supplement: Supplementary file 1 — Supplementary Figure and Table [file 41598_2017_6744_MOESM1_ESM.pdf]

Supplementary Information

Tonoplast-localized nitrate uptake transporters involved in vacuolar nitrate efflux and reallocation in *Arabidopsis*

Ya-Ni He[1,2,3], Jia-Shi Peng[1], Yao Cai[1,2], De-Fen Liu[1,2], Yuan Guan[1,3], Hong-Ying Yi[1], Ji-Ming Gong[1,*]

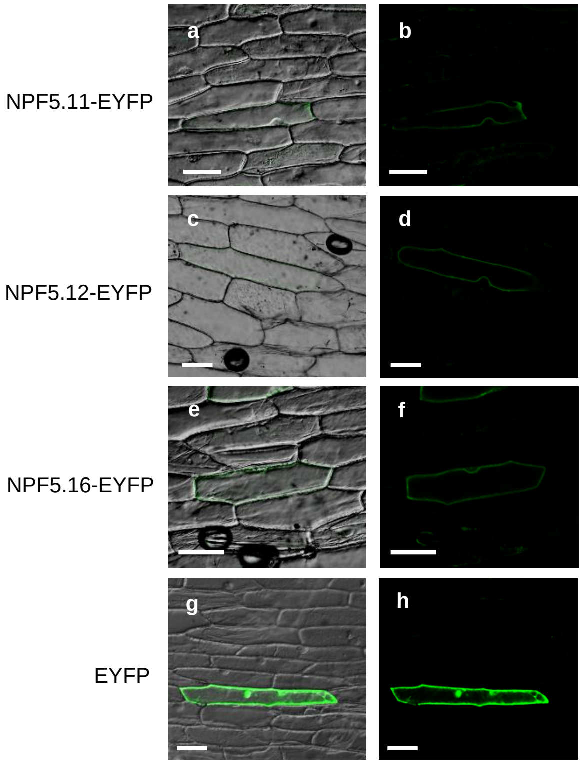

Supplementary Figure S1. Subcellular localization of NPF5.11, NPF5.12 and NPF5.16.

Onion epidermal cells transiently transformed with *NPF5.11-EYFP* (**a**,**b**), *NPF5.12-EYFP* (**c**,**d**), *NPF5.16-EYFP* (**e**,**f**) fusion or single *EYFP* (**g**,**h**) under the drive of cauliflower mosaic virus 35S promoter. Overlap images of EYFP fluorescence (green) and bright-field (**a**,**c**,**e**,**g**), EYFP fluorescence (**b**,**d**,**f**,**h**) images are shown. Bars = 100 μm.

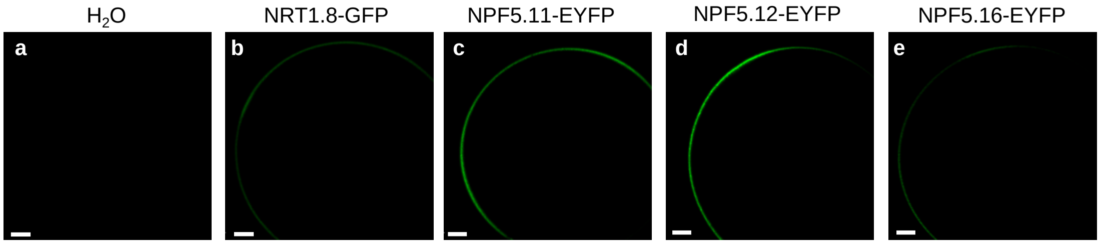

Supplementary Figure S2. Expression of *NPF5.11*, *NPF5.12* and *NPF5.16* in oocytes.

Oocytes were injected with H_2O (**a**) or cRNA of *NRT1.8-GFP* (**b**), *NPF5.11-EYFP* (**c**), *NPF5.12-EYFP* (**d**), *NPF5.16-EYFP* (**e**) and imaged after incubation for 2 days. Bars = 100 μm.

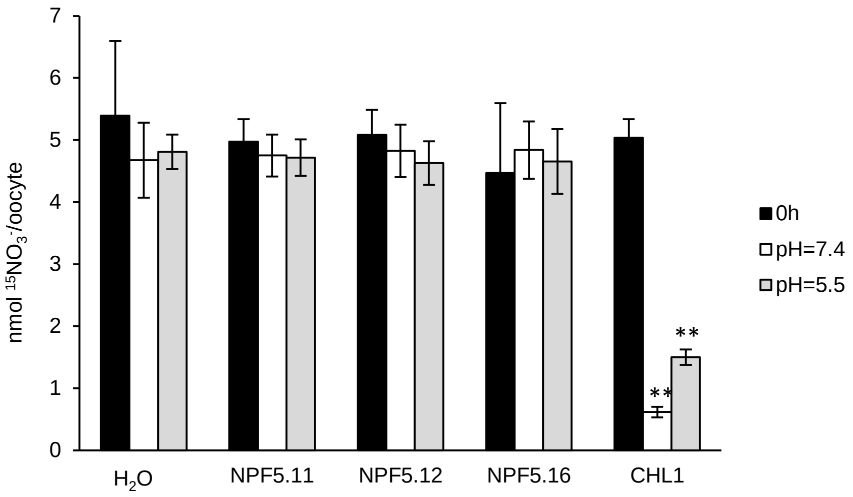

Supplementary Figure S3. NPF5.11, NPF5.12 and NPF5.16 could not export nitrate in oocytes.

50 nL of $K^{15}NO_3$ (100 mM) was injected into oocytes previously injected with water, NPF5.11-, NPF5.12-, NPF5.16- or CHL1-cRNA. At 0 h (immediately after injection) or after 3 h of incubation in ND96 buffer at pH 5.5 or 7.4, oocytes were washed three times with ND96 buffer and ^{15}N was determined. Values are means ± SD (n = 6-10). Asterisks indicate difference at $P < 0.01$ (**) compared with 0 h by Student's *t*-test.

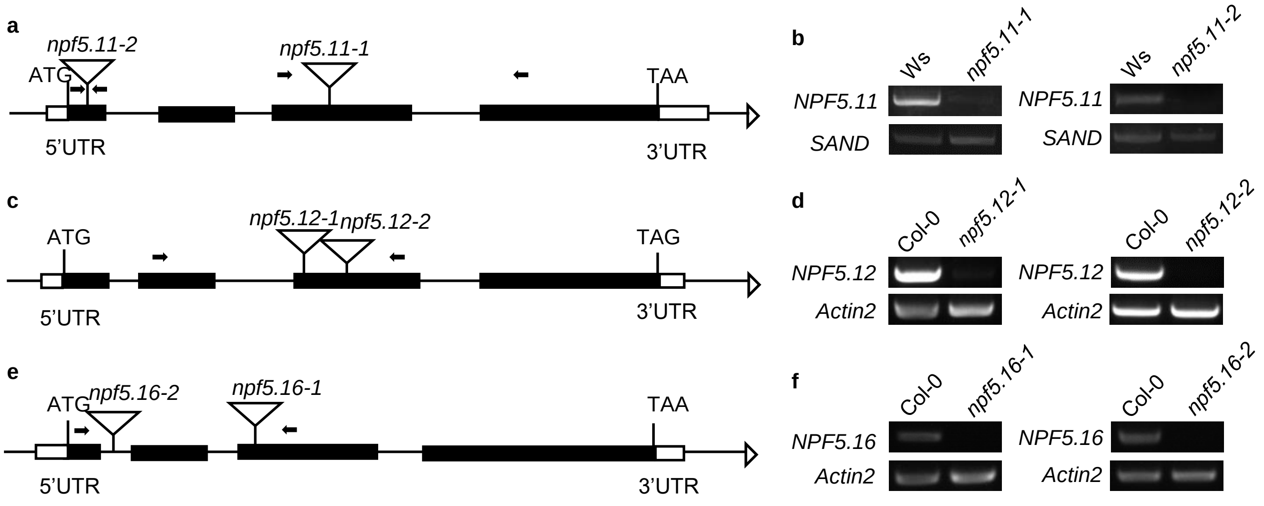

Supplementary Figure S4. The identification of *npf5.11*, *npf5.12* and *npf5.16* knock-out mutants.

(**a**,**c**,**e**) Schematic map of the T-DNA insertion sites in *npf5.11* (**a**), *npf5.12* (**c**) or *npf5.16* (**e**). Black boxes, coding regions; white boxes, untranslated regions (UTRs); Arrows, primers used for RT-PCR. (**b**,**d**,**f**) Expression of *NPF5.11* (**b**), *NPF5.12* (**d**) and *NPF5.16* (**f**) in the corresponding mutant lines, respectively. RNA isolated from roots (**d**) or leaves (**b**,**f**) of 28-d-old plants grown in hydroponics was used for RT-PCR analysis. *ACTIN* or *SAND* (loading control) was amplified for 28 cycles, *NPF5.11* (**b**) was amplified for 32 cycles, *NPF5.12* (**d**) and *NPF5.16* (**f**) were amplified for 35 cycles.

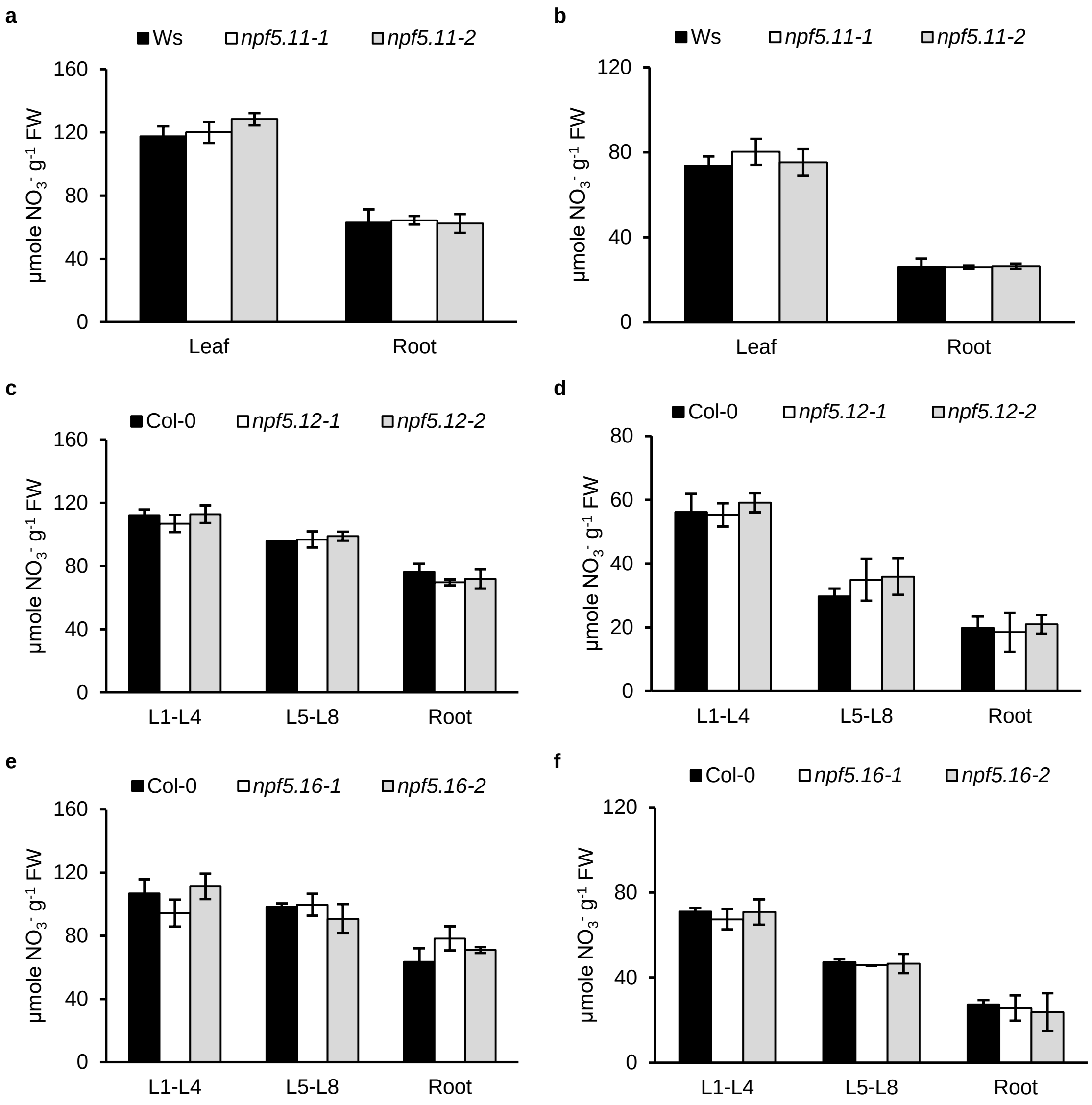

Supplementary Figure S5. Mutation of *NPF5.11*, *NPF5.12* or *NPF5.16* do not affect nitrate accumulation under both control and nitrogen-starved conditions.

(**a**,**b**) Plants were cultivated in hydroponics for 24 d (**a**) and then were treated with nitrogen-starved nutrient solution for 1 d (**b**), the leaves and roots of Ws and *npf5.11* were harvested to analyze nitrate concentration. Values are means ± SD, n = 3. (**c**,**d**) Plants were cultivated in hydroponics for 28 d (**c**) and then were treated with nitrogen-starved nutrient solution for 1 d (**d**), old leaves (L1-L4), young leaves (L5-L8) and roots of Col-0 and *npf5.12* were harvested to analyze nitrate concentration. Values are means ± SD, n = 3. (**e**,**f**) Plants were cultivated in hydroponics for 28 d (**e**) and then were treated with nitrogen-starved nutrient solution for 1 d (**f**), old leaves (L1-L4), young leaves (L5-L8) and roots of Col-0 and *npf5.16* were harvested to analyze nitrate concentration. Values are means ± SD, n = 3.

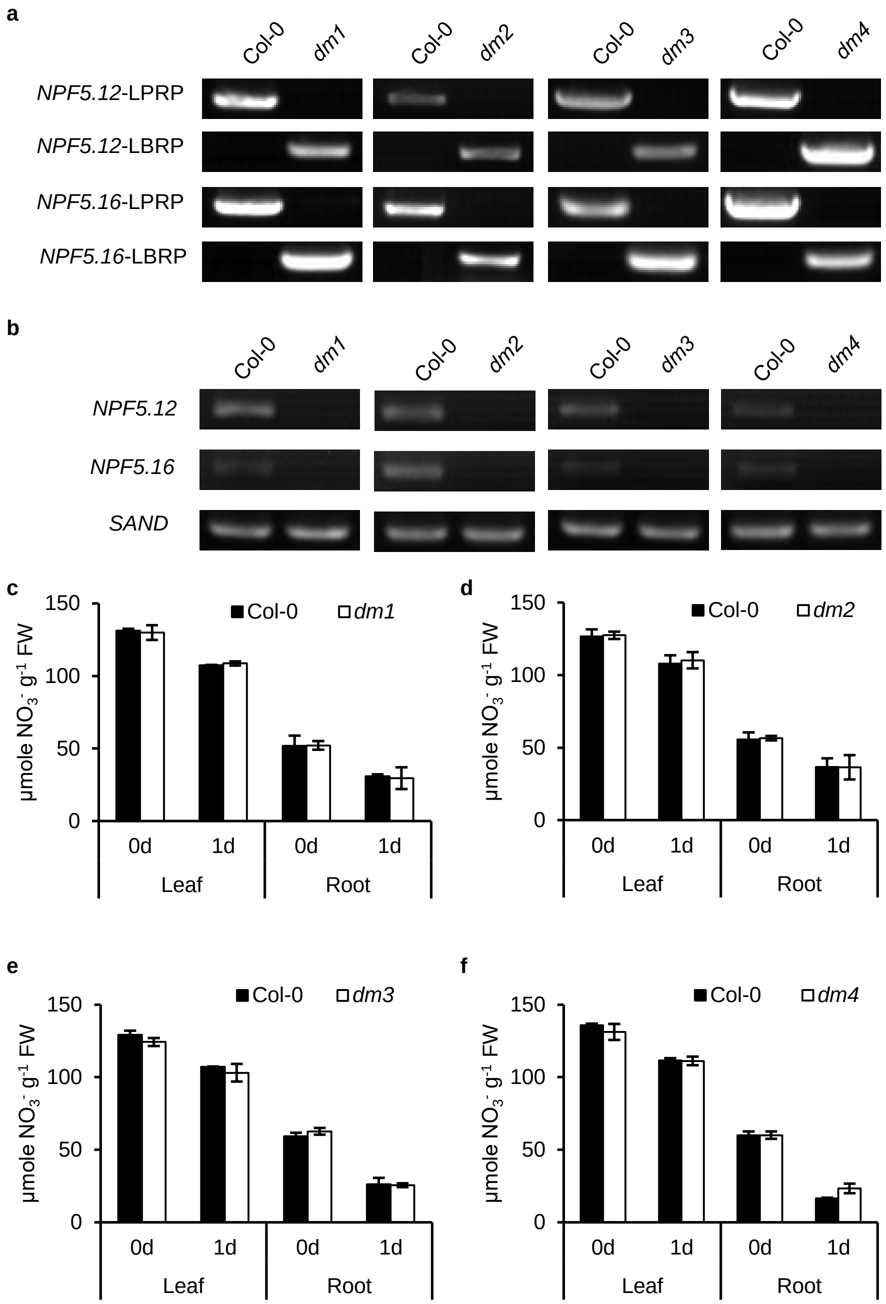

Supplementary Figure S6. Nitrate contents is not affected in *npf5.12 npf5.16* double mutant lines.

(**a**,**b**) Analysis of T-DNA insertion (**a**) and *NPF5.12*, *NPF5.16* expression in seedlings of *npf5.12 npf5.16* double mutant lines (**b**). *dm1*, *npf5.12-1 npf5.16-1*; *dm2*, *npf5.12-1 npf5.16-2*; *dm3*, *npf5.12-2 npf5.16-1*; *dm4*, *npf5.12-2 npf5.16-2*. *SAND* (loading control) was amplified for 28 cycles, *NPF5.12* and *NPF5.16* was amplified for 32 cycles in (**b**). (**c**-**f**) Nitrate concentration in *dm1* (**c**), *dm2* (**d**), *dm3* (**e**), *dm4* (**f**). 23-day-old plants were subjected to nitrogen-starved nutrient solution for indicated time. The leaves and roots of wild type and double mutant lines were harvested to analyze nitrate concentration. Values are means ± SD, n = 3.

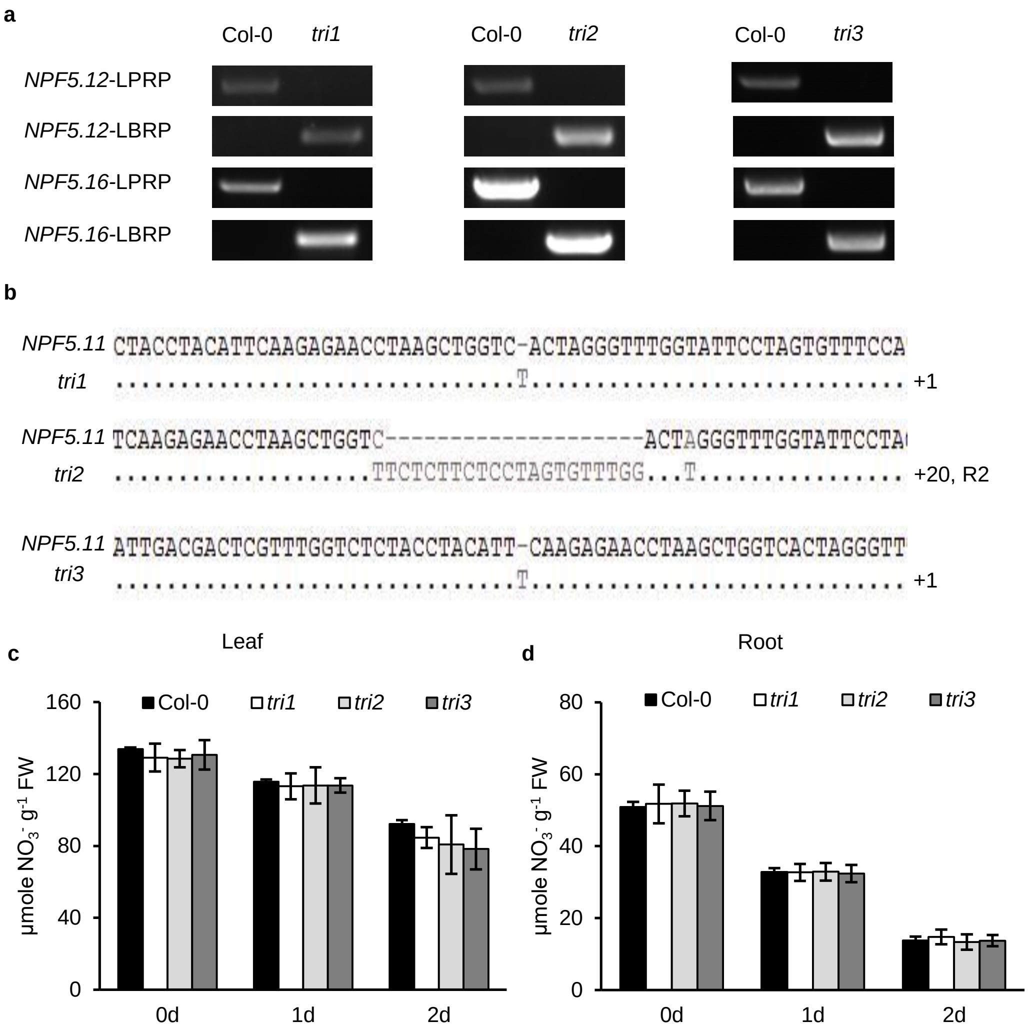

Supplementary Figure S7. Nitrate contents is not affected in *npf5.11 npf5.12 npf5.16* triple mutant lines.

(**a**) PCR identification of T-DNA insertion in *npf5.11 npf5.12 npf5.16* triple mutant lines. (**b**) Sequence of edited *NPF5.11* in triple mutant lines. The nucleotide changes were on the right of each sequence (+, insertion; R, replace). (**c**,**d**) Nitrate concentration in leaves (**c**) or roots (**d**) of triple mutant lines. 28-day-old plants were subjected to nitrogen-starved nutrient solution for indicated time. Values are means ± SD, n = 3.

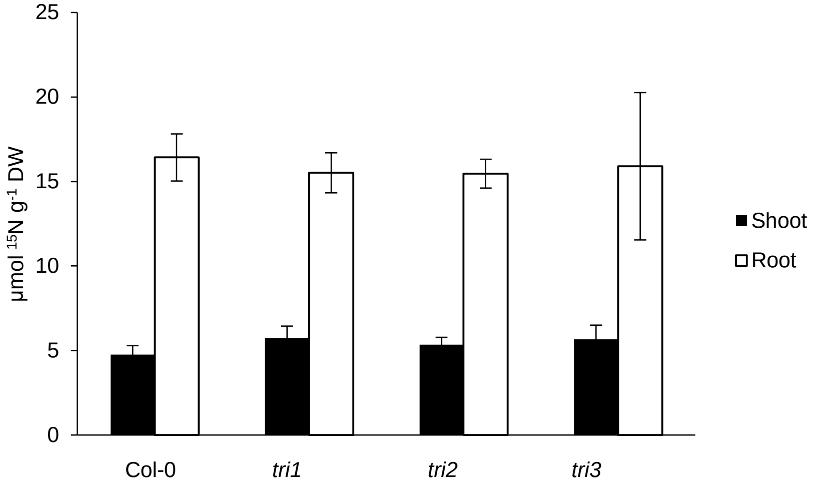

Supplementary Figure S8. $^{15}NO_3^-$ concentration in shoots and roots of triple mutant plants.

Plants were grown in hydroponics for 28 days and treated with 2.25 mM $K^{15}NO_3$ for 30 min. ^{15}N concentration in shoots and roots were analyzed. Values are means ± SD, n = 3.

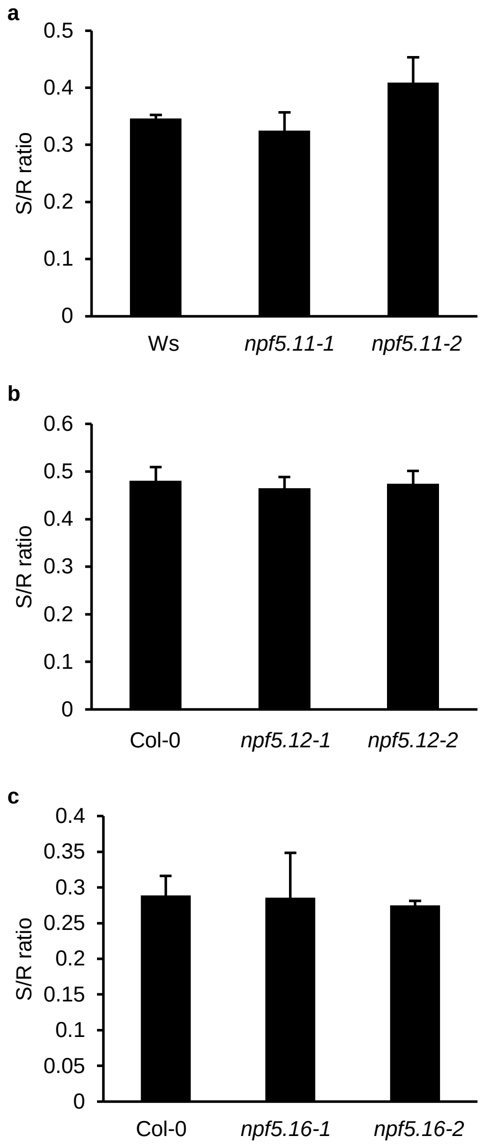

Supplementary Figure S9. Root-to-shoot nitrate transport was not affected in *npf5.11*, *npf5.12*, *npf5.16* single mutants.

28-day-old plants were treated with 2.25 mM $K^{15}NO_3$ for 30 min, ^{15}N contents in shoots and roots were detected and the ^{15}N concentration ratio in shoot and root (S/R ratio) of *npf5.11* (**a**), *npf5.12* (**b**), *npf5.16* (**c**) were calculated. Values are means ± SD, n = 3.

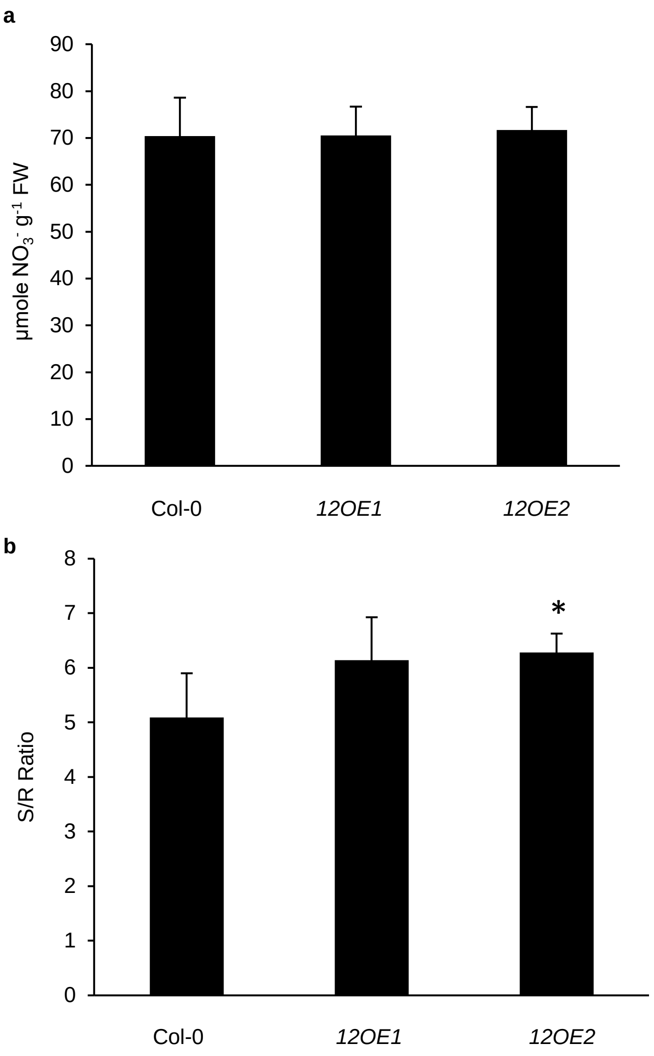

Supplementary Figure S10. Nitrate accumulation in shoots and the S/R ratio of *NPF5.12* overexpression lines.

24 days old plants grown hydroponically were subjected to nitrogen-starvation for 30 h, then nitrate contents in shoots were determined by HPLC (**a**) and the S/R ratios were calculated (**b**). *12OE1* and *12OE2* were two independent *NPF5.12* overexpression lines. Values are means ± SD, n = 5-7. Asterisks indicate difference between wild type and overexpression lines at $P<0.05$ (*) by Student's t-test.

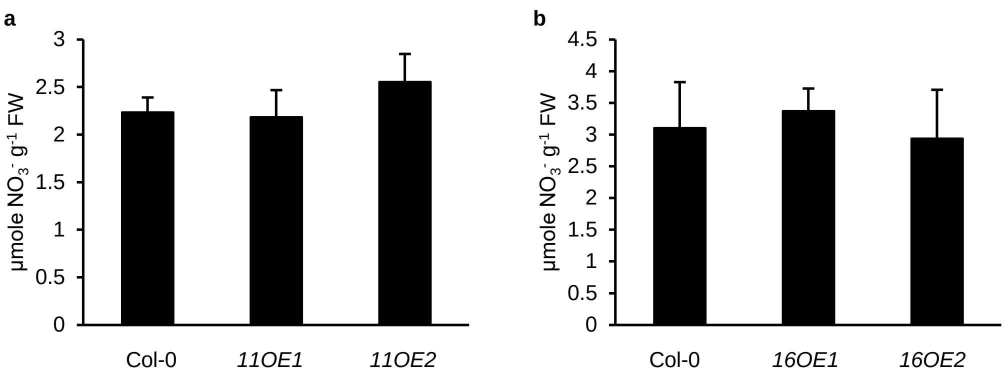

Supplementary Figure S11. Overexpression of *NPF5.11* or *NPF5.16* do not affect nitrate contents in roots under nitrogen-starved condition.

28 days old hydroponically grown plants were subjected to nitrogen-starvation for 2 d, then roots of *11OE1* and *11OE2* (**a**), *16OE1* and *16OE2* (**b**) were sampled and nitrate contents were measured by HPLC. *11OE1* and *11OE2* were two independent *NPF5.11* overexpression lines. *16OE1* and *16OE2* were two independent *NPF5.16* overexpression lines. Values are means ± SD, n = 3.

Supplemental Table S1. List of primer sequences

Destination	Forward primer	Reverse primer
**Genotyping**		
npf5.11-1	CTGAGGGTTTTCTGGAAATCC	CTGCCCTGTCTAGGAATCTGC
npf5.11-2	GTATGAATGTACGGCCAGTGC	TCCCTGTTTGTTTTGTTTTCG
npf5.12-1	CAAACGTTAACCTCTGGCTTG	TTTTGTTTGTAAACAAAAACACAGTG
npf5.12-2	CAAACGTTAACCTCTGGCTTG	TTTTCATCTCGACCAAAGCAG
npf5.16-1	TCGTGTTCATTATTCACGCAG	GAAAACTCTACCGATCCTCGC
npf5.16-2	CGCCTTGTTTTGTGAAGAAAG	TCGTGTTCATTATTCACGCAG
LB4	CGTGTGCCAGGTGCCCACGGAATAGT	
GABI-LB	ATATTGACCATCATACTCATTGC	
LB3	TAGCATCTGAATTTCATAACCAATCTCGATACAC	
LBb1.3	ATTTTGCCGATTTCGGAAC	
**CRISPR-Cas9 system**		
Target sequence-1	GAGAACCTAAGCTGGTCACT	
Target sequence-2	GCTTAGGTTCTCTTGAATGT	
Cas9	AGCAGCCGACAAGAAGTACAGC	GCTTTCAGCAGGGTCAGGTCCT
**Transient expression & Plant transformation**		
NPF5.11	ctcgagATGGCTATCACCTACTCCTC	actagtAAAGGTGTTTGATCTGCTGT
NPF5.12	ggatccATGTCGACATCCATCGGCGATA	actagtCTTTGGGCTGTTGTAGAGAT
NPF5.16	gtcgacATGGCGATAGCCGAAGA	ggatccGACTTGATCTACACGGC
*NPF5.11*pro	gtcgacTACAGCTCTTGTCAGGCTTA	ggatccTAATGTGTATGATTGATTGG
*NPF5.12*pro	gtcgacCTAACGCGACAGCAAGCACT	ggatccTGCTTCTTGTTATTTCGTTT
*NPF5.16*pro	gtcgacCATTGCGCGGTGGGCAAT	cccgggTTTCTTGTCGGAGGAAAC
**RT-PCR & qRT-PCR**		
npf5.11-1	CGAATCAGCTGCAAGAGACTATCT	AAGAGTCCTGGTGAGATTGACCTG
npf5.11-2	ATGGCTATCACCTACTCCTCCGC	GATGATGAGCCTCGCTGATTTCC
npf5.12-1/*npf5.12-2*	AATCTGATAACCTACTTCACCGAGGC	CGGTAAGTTTTGATTCCAAGTAAGAACAAG
npf5.16-1/*npf5.16-2*	GATAGCCGAAGAAGAAGCTGCA	CCAAAAGCTTGAACACATGGCT
Actin2	CCCTGTTCTTCTTACCGAG	CCACATCTGCTGGAATG
SAND	GATGAGGATGATGCTTCTACG	CCTGAGCGTTGTATCTTGGT
q*NPF5.11*	GCGTTGGTATGGCTTTGAAC	GCCAGTAGCCAGTAGAAGTAATC
q*NPF5.12*	CATGGTATCAGTCATCGAGGAAG	GACAAGCAAGTAGCCAGTAGAA
q*NPF5.16*	CCACGGAGCTAAGGAGTATTG	GGGCTCTGTTCAGGTTAGTATT
q*SAND*	ATATGACACCCTTGCTTGGAGGGA	TGAGAATAAGACACCAGACGCGCA
